# Rat Locomotion Detection Based on Brain Functional Directed Connectivity from Implanted Electroencephalography Signals

**DOI:** 10.3390/brainsci11030345

**Published:** 2021-03-09

**Authors:** Bo Li, Minjian Zhang, Yafei Liu, Dingyin Hu, Juan Zhao, Rongyu Tang, Yiran Lang, Jiping He

**Affiliations:** 1 School of Mechatronical Engineering, Beijing Institute of Technology, Beijing 100081, China; 3120170125@bit.edu.cn (B.L.); 3120170166@bit.edu.cn (M.Z.); yafei.liu@bit.edu.cn (Y.L.); dy@bit.edu.cn (D.H.); jiping.he@bit.edu.cn (J.H.); 2 Beijing Innovation Centre for Intelligent Robots and Systems, Beijing Institute of Technology, Beijing 100081, China; tangrongyu@bit.edu.cn; 3 Department of Materials Processing Engineering, School of Materials Science and Engineering, Beijing Institute of Technology, Beijing 100081, China; 7520190072@bit.edu.cn

**Keywords:** electroencephalography, locomotion detection, granger causality, brain functional directed connectivity, freely walking rats, machine learning

## Abstract

Previous findings have suggested that the cortex involved in walking control in freely locomotion rats. Moreover, the spectral characteristics of cortical activity showed significant differences in different walking conditions. However, whether brain connectivity presents a significant difference during rats walking under different behavior conditions has yet to be verified. Similarly, whether brain connectivity can be used in locomotion detection remains unknown. To address those concerns, we recorded locomotion and implanted electroencephalography signals in freely moving rats performing two kinds of task conditions (upslope and downslope walking). The Granger causality method was used to determine brain functional directed connectivity in rats during these processes. Machine learning algorithms were then used to categorize the two walking states, based on functional directed connectivity. We found significant differences in brain functional directed connectivity varied between upslope and downslope walking. Moreover, locomotion detection based on brain connectivity achieved the highest accuracy (91.45%), sensitivity (90.93%), specificity (91.3%), and F1-score (91.43%). Specifically, the classification results indicated that connectivity features in the high gamma band contained the most discriminative information with respect to locomotion detection in rats, with the support vector machine classifier exhibiting the most efficient performance. Our study not only suggests that brain functional directed connectivity in rats showed significant differences in various behavioral contexts but also proposed a method for classifying the locomotion states of rat walking, based on brain functional directed connectivity. These findings elucidate the characteristics of neural information interaction between various cortical areas in freely walking rats.

## 1. Introduction

Brain connectivity effectively describes information flow (including direction and strength) between cortical areas [1], which comprises brain anatomical structure or functional associations [2]. Brain connectivity has three forms: structural connectivity, functional connectivity, and effective connectivity [3]. Structural connectivity represents the anatomical connectivity of various brain regions [4]; functional connectivity describes the temporal dependency of separate brain areas [5]; and effective connectivity reflects causal interactions between different brain regions in a directly manner [6]. With the continuous development of brain network analysis technology, brain connectivity estimation has been widely used in neuroscience research, including disease diagnosis [7,8], emotional state recognition [9], as well as action and intention recognition in brain–computer interfaces [10,11,12]. Post-traumatic stress disorder and major depression have significant difference within the frontoparietal network and the default mode network, and based on these differences in connectivity, two clinically relevant subtypes have been successfully identified [8]. An emotional classification system using brain connectivity and convolutional neural networks has been introduced, achieving excellent classification performance [9].

Previous studies have revealed that the cortex plays a critical role in walking control in freely walking rats [13,14] instead of relying solely on spinal cord motor networks. Further, the extent of cortex modulations appeared linked to the degree of volitional engagement and the complexity of locomotion [13]. The spectral characteristics of electrocortical activity in various brain regions also presented significant differences in behavioral conditions [14]. However, whether brain connectivity across distributed brain regions show significant differences during walking tasks in freely moving rats remains undetermined. Further, whether the brain connectivity can be used to detect the locomotion states is yet unknown.

In rodent models, brain network analysis is mainly used to study changes in connectivity during the evolution of diseases, such as epilepsy [15], depression [16], Huntington’s disease [17], and Alzheimer’s disease [18]. Studies on brain connectivity related to locomotion mainly focus on humans, and network analysis has not been applied to explore the principles of rodent locomotion. Significant differences in brain connectivity in humans under different motion states have been indicated in several reports. Standing has stronger connections in sensorimotor areas than walking. The opposite happens in non-sensorimotor areas (prefrontal cortex, posterior parietal, and anterior cingulate) when subjects are simultaneously engaged in a cognitive task [19]. Similarly, compared with corresponding networks of normal walking, the supplementary motor and prefrontal areas exhibit greater connectivity associated with walking while talking [20]. Notably, significant changes in brain connectivity during motion preparation and execution were observed. During the execution of the foot movement, the cingulate motor areas work as network hubs, revealing numerous outgoing edges to other regions. During foot movement preparation, the brain networks exhibited the highest global efficiency and stronger small world attributes [21]. A study also showed that global brain connectivity decreased in people with stroke relative to healthy ones. The effects were particularly larger during pedaling than foot-tapping. Moreover, local brain connectivity was higher in stroke than healthy participants only during paretic foot tapping [22].

As earlier described, our knowledge of brain connectivity properties in rats performing walking locomotion tasks remains limited despite the wise use of rodent models in neuroscience. This study aimed to investigate differences in the brain connectivity of rats in different walking locomotion states. To address this problem, we recorded the locomotion states and implanted electroencephalography (EEG) signals in freely moving rats performing two kinds of natural task conditions (upslope and downslope walking). Although functional connectivity can measure the temporal synchronization correlation between active brain regions, effective connectivity indicating how the activity of one region influences other separate regions provides more information, to be more suitable to explore the variability of the brain connectivity in freely walking rats. Currently, in neuroscience, Granger causality is the most popular algorithm for extracting directional transmission information between brain regions [23]. It can effectively measure the effective connectivity among neural signal sources in the brain. Thus, in this study, we applied the Granger causality algorithm to determine brain connectivity in rats during walking under different behavior conditions. Three kinds of classifiers—k-nearest neighbors (KNN), random forest (RF), and support vector machine (SVM)—were then applied to categorize the two walking states on the basis of brain connectivity. We predicted an evident difference in brain connectivity when rats performed different walking tasks. Based on this difference, motion states could be classified using machine learning algorithms.

## 2. Materials and Methods

### 2.1. EEG Data Description

**Figure 1:**
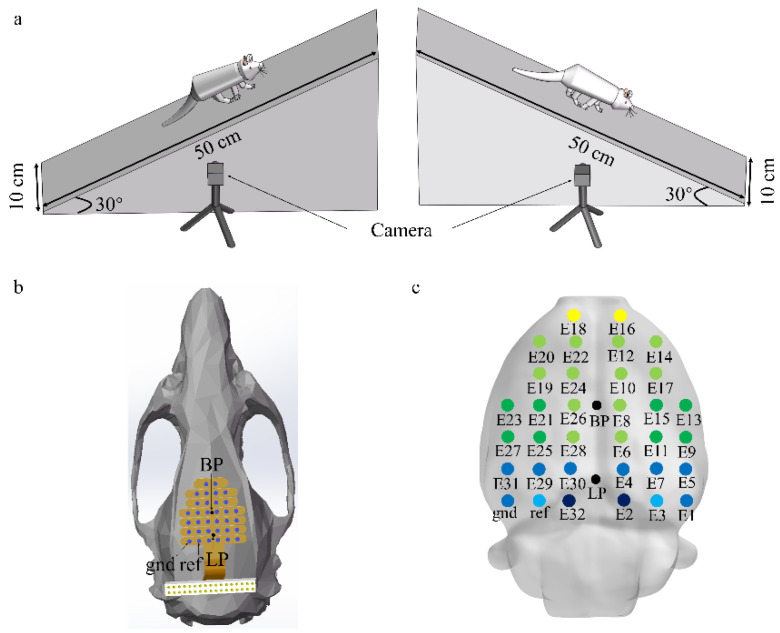
Recording setup and task conditions. (**a**) Characteristics of locomotor paradigms (upslope and downslope walking). (**b**) Electrode location on the rat skull surface; blue dots representing screws that attach the electrode array to the skull; BP represented the bregma point; LP represented lambda point. (**c**) Putative electrode positions on the rat brain, obtained using Brainstorm3. The color of the electrode corresponds to the underlying brain region (yellow: frontal cortex; light green: somatomotor areas; green: somatosensory areas; light blue: visual cortex; blue: posterior parietal association area; dark blue: retrosplenial area).

In this study, previous EEG data on four male Sprague–Dawley rats (aged 8–10 weeks and weighing 200–250 g) were used to examine differences in brain connectivity in rats in different locomotion states [14]. Two types of locomotion tasks (upslope and downslope walking) were performed by the rats in the experiment. The task conditions are shown in Figure 1a. Simultaneously, a 32-channel invasive EEG electrode array was fixed on the skull to record the EEG data of the rats, and the rat locomotion states were recorded by two cameras (80 Hz) (see reference [14] for surgical details). Figure 1b shows the implantation of the electrode array on the skull. The screws penetrated the skull to reach the epidural space. Figure 1c shows the putative electrode positions on the rat brain surface, obtained using the software Brainstorm3. The sampling rate of the EEG data is 1000 Hz.

### 2.2. EEG Data Preprocessing

Preprocessing of EEG data was performed using EEGLAB 14.1.2b functions [24]. First, the EEG data were screened with a band-pass filter at 7–100 Hz and a notch filter at 50 Hz by using the EEGLAB function “eegfilt” (zero-phase finite impulse response filter, order 1100). Second, the function “common average” was used to re-reference the EEG data. Third, the EEG data were parsed using the ICA algorithm to get independent component (IC) sources. By visually inspecting each IC scalp projection and power spectrum, we identified and removed those IC sources related to no-brain artifacts (head shaking artifacts) to obtain clean EEG data. Fourth, the EEG data were divided by locomotion states into epochs, which started the moment of rat paw first touched the slope and ended the moment of the same paw next contacted the slope. The average length of each epoch was 600 ms. The epochs were rejected if they contained values exceeding the average of the probability distribution of values across the data segments by 5 standard deviations [25]. Approximately 1% of epochs were eliminated in per condition across all animals.

Ultimately, to better explore the difference in brain effective connectivity in rats walking on different locomotion terrains, the EEG data were divided into four major sub-bands: the alpha band (7–13 Hz), beta band (13–30 Hz), low gamma band (30–50 Hz), and high gamma band (50–100 Hz). The filter used in this study is zero-phase finite impulse response filter in EEGLAB, whose design method is two-way least-squares. The present study investigated variations in brain connectivity in the full-frequency band (7–100 Hz) and four sub-band signals.

### 2.3. Functional Directed Connectivity Computation

The Granger causality algorithm was utilized to compute brain connectivity in this study. Notably, our brain connectivity analysis mainly discussed functional directed characteristics. Therefore, in this study, the brain connectivity is more appropriately called “functional directed connectivity” rather than “effective connectivity”. The electrodes on the rat skull surface acted as nodes of connectivity EEG signals recorded by each electrode reflects the activities of neurons in the brain area below and nearby [26]. Thus, the brain functional directed connectivity reflected the dependency relationship between different activated brain regions [27]. Based on definition, Granger causality is a widely used statistical method for parsing the dependency relationship between two signals [28]. The definition of Granger causality is that if the past information of variables *x* and *y* is included, the prediction of *y* is better than the prediction of *y* solely based on the past information of *y*. That is, if the variable *x* can help predict the future variable *y*, the variable *x* is considered to be the “Granger cause” of the variable *y* [28]. In the present study, the Granger causality was applied to obtain the connectivity between all electrode pairs. The mathematical derivation process is as follows: *x*(*t*) and *y*(*t*) respectively represent the EEG signals from the two electrodes. They can be described as the following formula according the bivariate autoregressive model [29,30]:

(1) $$x\left(t\right)=\overset{p}{\underset{k=1}{\sum }}a_{x|x,k}x\left(t-k\right)+\overset{p}{\underset{k=1}{\sum }}a_{x|y,k}y\left(t-k\right)+u_{xy}\left(t\right)$$

(2) $$y\left(t\right)=\overset{p}{\underset{k=1}{\sum }}a_{y|x,k}x\left(t-k\right)+\overset{p}{\underset{k=1}{\sum }}a_{y|y,k}y\left(t-k\right)+u_{yx}\left(t\right)$$

where the coefficient a are the model parameters, *p* is the order, and *u_xy_*(*t*) and *u_yx_*(*t*) are the residuals associated with the models of *x*(*t*) and *y*(*t*), respectively. In this study, the selection of *p* is based on the AIC criteria and the validation of the bivariate autoregressive model is based on the Ljung-Box test. 

The residuals then depend on the past values of both signals, and their variances are

(3) $$V_{x|\bar{x,}\bar{y}}=var\left(u_{xy}\right)$$

(4) $$V_{y|\bar{x,}\bar{y}}=var\left(u_{yx}\right)$$

where *var*(.) is the variance over time, and *x*|*x*,*y* is the prediction of *x*(*t*) by the past samples of values of *x*(*t*) and *y*(*t*).

Therefore, Granger causality from *y* to *x* (predicting *x* from *y*) is

(5) $$GC_{y\to x}=\ln\left(\frac{V_{x|\bar{x}}}{V_{x|\bar{x,}\bar{y}}}\right)$$

**Figure 2:**
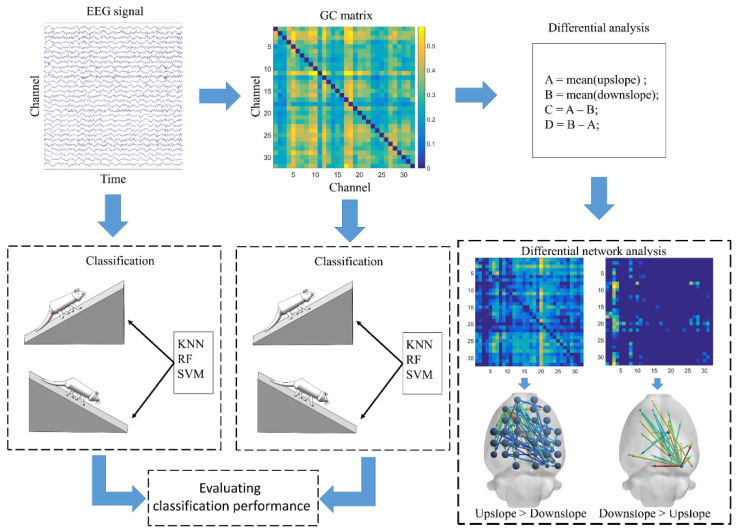
Schematic of electroencephalography (EEG) data processing flow.

In the current study, the Hermes toolbox was used to calculate the matrix formed by the brain functional directed connectivity, based on its built-in Granger causality algorithm from EEG data [30]. The obtained connectivity matrix performed two types of analysis. One was to analyze the difference in brain functional directed connectivity between upslope and downslope walking. The other was to classify the uphill and downhill motion states, based on connectivity features, by machine learning. Simultaneously, in evaluating the classification performance, we used EEG signals to classify the motion states. Figure 2 presents a schematic of the EEG data processing flow. Connectivity matrix measurement was performed in the Brain Connectivity Toolbox. The clustering coefficient is a vital parameter to measure network characteristics, which measure the inherent tendency to cluster of network [31]. In this study, we used the clustering coefficient measure to reflect the clustering tendency of each node, to measure the importance of one node in the neural information interaction with others. The BrainNet Viewer toolbox was used to visualize the brain functional directed connectivity [32].

### 2.4. Machine Learning Algorithms for Classifying Locomotion States by Brain Functional Directed Connectivity

In this study, three kinds of popular classifiers were used in locomotion state detection: KNN, RF, and SVM. KNN classifies samples based on measuring the distance between different feature values [33]. The core idea is that one sample belongs to the category of the k samples that are most similar to it. That is, the classification decision of this method is based on the category of the nearest one or several samples to determine the category of the sample to be classified. RF is an algorithm that integrates multiple decision trees through the idea of ensemble learning [34]. The classification performance of RF depends on the performance of a single decision tree and the correlation between them. During the training process, each decision tree will randomly and put back training samples from the training set. The basic idea of SVM is to solve the separation hyperplane that can correctly divide the training data set and maximize the geometric interval [35]. As a parametric technique, SVM transforms nonlinearity into linearity [36].

We calculated the functional directed connectivity matrix between all pairs of electrodes (32 × 32 = 1024 connections), obtaining 1024 connectivity metrics per locomotion state. The strength of the connection between an electrode and itself was zero and thus could not provide effective features for the classifier. Consequently, we removed them from the connectivity metrics, finally obtaining 992 (1024−32 = 992 connections) dimensional classification features. In this study, the dimension of EEG features was 19,200 (32 × 600 = 19,200). 

In this study, we selected four evaluation metrics (accuracy, sensitivity, specificity, and F1-scores) widely used in machine learning, to evaluate the detection effect of rats locomotion states [37]. Accuracy (ACC) is defined as the proportion of correctly classified samples to the total samples. Sensitivity, namely, true positive ratio (TPR), is defined as the proportion of the number of true positives (TP) to the sum of the TP and false negatives (FN). Specificity, also called true negative rate (TNR), is defined as the proportion of true negatives (TN) to the sum of false positive (FP) and TN. F1-score is the harmonic mean of precision and sensitivity, where precision is the proportion of TP to the sum of TP and FN. F1-score combines sensitivity and accuracy, showing a more comprehensive evaluation index. In this study, we classified the detection results according F1-score, perfect (F1-scores >90%), high (F1-scores >85% and <90%), satisfactory (F1-scores >80% and <85%), and not satisfactory (F1-scores <80%).

### 2.5. Statistics

Linear mixed-effects models (using the MATLAB function “fitlme”) were used to test for the presence of significant differences in brain functional directed connectivity for different locomotion states. Because the connectivity measures that comes from different rats are independent non-identically distributions, they are not suitable for the General Linear Model. The linear mixed-effects model can only consider fixed effects and remove random effects [38]. Therefore, the linear mixed-effects model can be used to calculate the significant differences in brain connectivity for different locomotion states, excluding the influence caused by different rats. All values are reported as mean ± 95% confidence interval values, unless otherwise specified. 

## 3. Results

### 3.1. Brain Functional Directed Connectivity in Rats Varied with the Locomotion State

**Figure 3:**
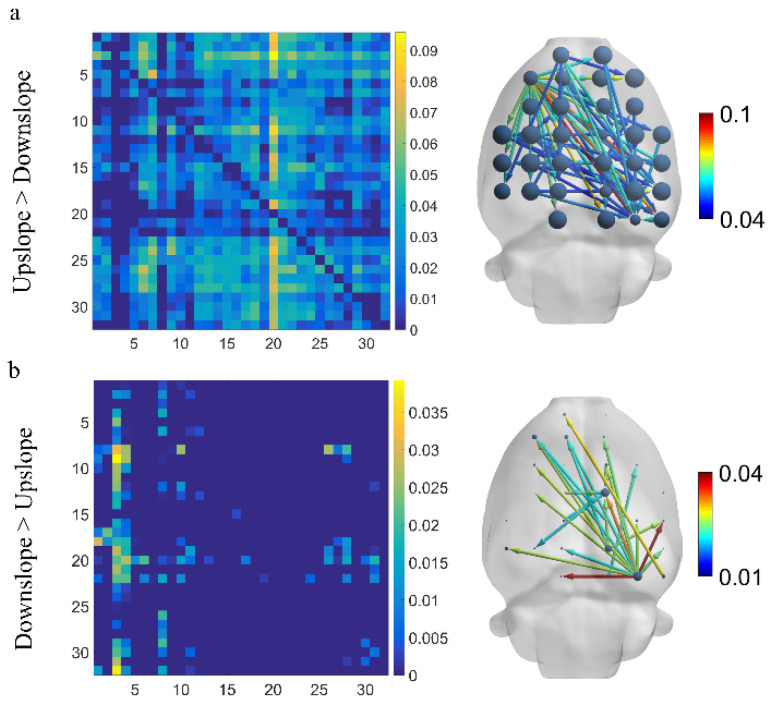
Difference in brain functional directed connectivity for two locomotion states in the full-frequency band (7–100 Hz). (**a**) Increased connectivity in upslope locomotion than downslope locomotion. (**b**) Increased connectivity in downslope locomotion than upslope locomotion. Threshold set to 50% of the strongest connection (i.e., preserving connections exceeding 50% of the strongest connection). The node size denotes the degree, and the edge color represents the connection weight.

We first identified variations in brain functional directed connectivity in the full-frequency band (7–100 Hz) in rats walking on different terrains. We found a significant difference in functional directed connectivity between different locomotion states. Specifically, compared with downslope-walking, the connectivity increased significantly when rats in upslope-walking, including larger-degree nodes, more connected edges, and greater connection weights (Figure 3a). The most greatly increased connections started from the left motor cortex to other brain areas. Only a small number of connections increased in rats walking on a downward slope relative to those walking on an upward slope (Figure 3b). The increased connections mainly originated from the right visual cortex to other brain regions.

**Figure 4:**
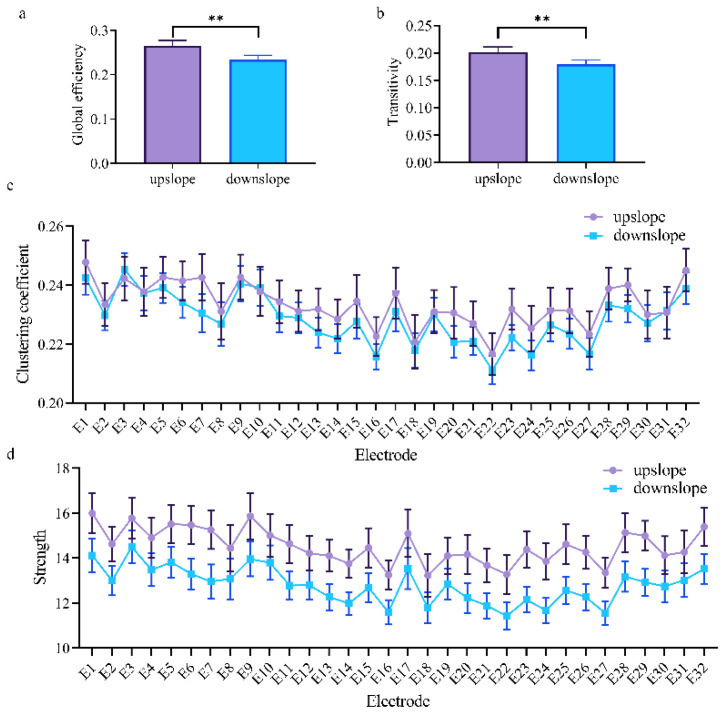
Indexes of the brain network for two locomotion states in the full-frequency band (7–100 Hz). (**a**,**b**) Global indexes of the brain network, including global efficiency and transitivity. (**c**,**d**) Local indexes of the brain network, including clustering coefficient and strength. All error bars indicate the 95% confidence interval of the mean. ** *p* ≤ 0.01.

At the network level, the brain network indexes showed a considerable difference between upslope and downslope walking. In upslope walking, the brain network exhibited an increased global efficiency (Figure 4a). Analogously, the transitivity of the brain network presented a similar trend for these two locomotion states (Figure 4b). This similarity indicated that the brain network exhibited a higher efficiency of information processing in rats walking on an upward slope. At the node level, a significant difference in connectivity index was also determined. The clustering coefficient and node strength were higher in rats walking on a downward slope than in those on a downward slope (Figure 4c,d). The efficiency of information processing in nodes in different brain regions evidently varied. The nodes located in the posterior parietal association cortex and retrosplenial area possessed higher clustering coefficients. That is, brain functional directed connectivity in rats varied with locomotion states in the full-frequency band.

### 3.2. Brain Functional Directed Connectivity in Rats Presented a Significant Difference in Each Sub-Band

**Figure 5:**
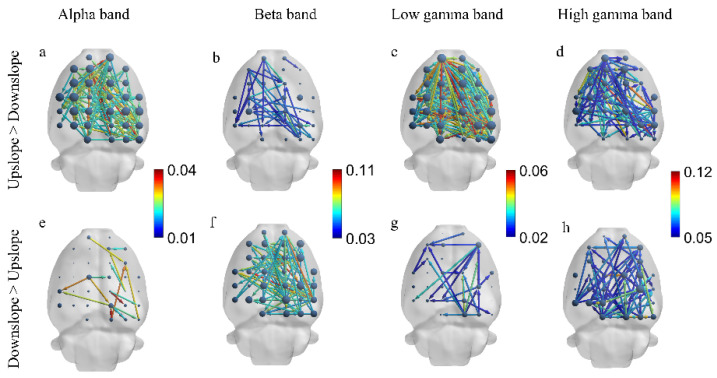
Difference in brain functional directed connectivity between two locomotion states in four sub-bands. (**a**) Increased connectivity in upslope locomotion relative to that in downslope locomotion in the alpha band (7–13 Hz). (**b**) Increased connectivity in upslope locomotion relative to that in downslope locomotion in the beta band (13–30 Hz). (**c**) Increased connectivity in upslope locomotion relative to that in downslope locomotion in the low gamma band (30–50 Hz). (**d**) Increased connectivity in upslope locomotion relative to that in downslope locomotion in the high gamma band (50–100 Hz). (**e**) Increased connectivity in the downslope relative to that in upslope locomotion in the alpha band (7–13 Hz). (**f**) Increased connectivity in downslope locomotion relative to that in upslope locomotion in the beta band (13–30 Hz). (**g**) Increased connectivity in downslope locomotion relative to that in upslope locomotion in the low gamma band (30–50 Hz). (**h**) Increased connectivity in downslope locomotion relative to that in upslope locomotion in the high gamma band (50–100 Hz). Threshold set to 50% of the strongest connection (i.e., preserving those connections exceeding 50% of the strongest connection). The node size is the degree, and the edge color denotes the connection weight.

We next explored the difference in brain functional directed connectivity in four major sub-bands—the alpha band (7–13 Hz), beta band (13–30 Hz), low gamma band (30–50 Hz), and high gamma band (50–100 Hz)—between rats in two locomotion states. Significant differences in brain functional directed connectivity for each sub-band were determined between upslope-walking and downslope-walking rats. In the alpha and low gamma bands, connections (including the node degree, connected edge, and connection weight) in the upslope state increased relative to those in the downslope state (Figure 5a,c); increased connections rarely occurred in the downslope state (Figure 5e,g). By contrast, in the beta band, connections in large numbers increased in the downslope state (Figure 5f), whereas few connections increased in the upslope state (Figure 5b). Notably, in the high gamma band, no significant difference in increased connected edges was found between the two locomotion states. However, stronger connections were mostly distributed in the left sensorimotor areas in the upslope-walking rats than in the downslope-walking ones. For the locomotion state of downslope walking, the stronger connections were mainly distributed in the right visual–motor integration areas (e.g., the retrosplenial area). 

**Figure 6:**
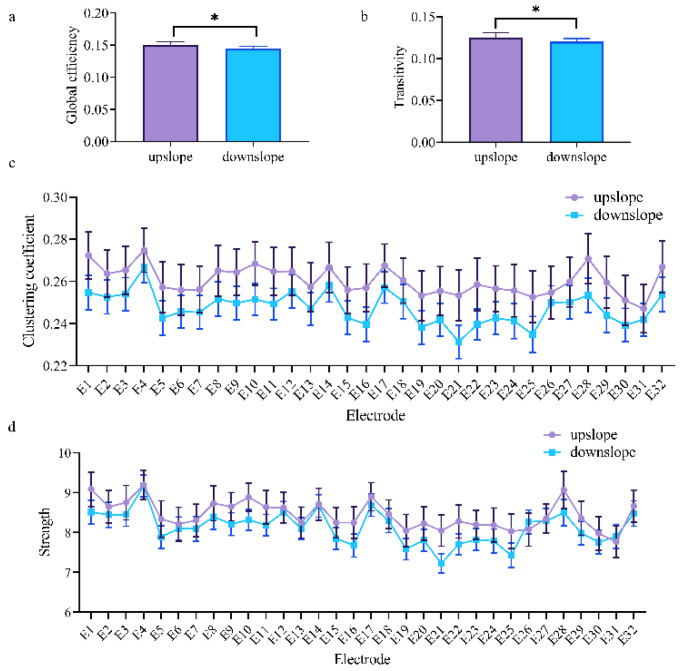
Indexes of the brain network for the two locomotion states in the alpha band (7–13 Hz). (**a**,**b**) Global indexes of the brain network, including global efficiency and transitivity. (**c**,**d**) Local indexes of the brain network, including clustering coefficient and strength. All error bars indicate the 95% confidence interval of the mean. * *p* < 0.05.

**Figure 7:**
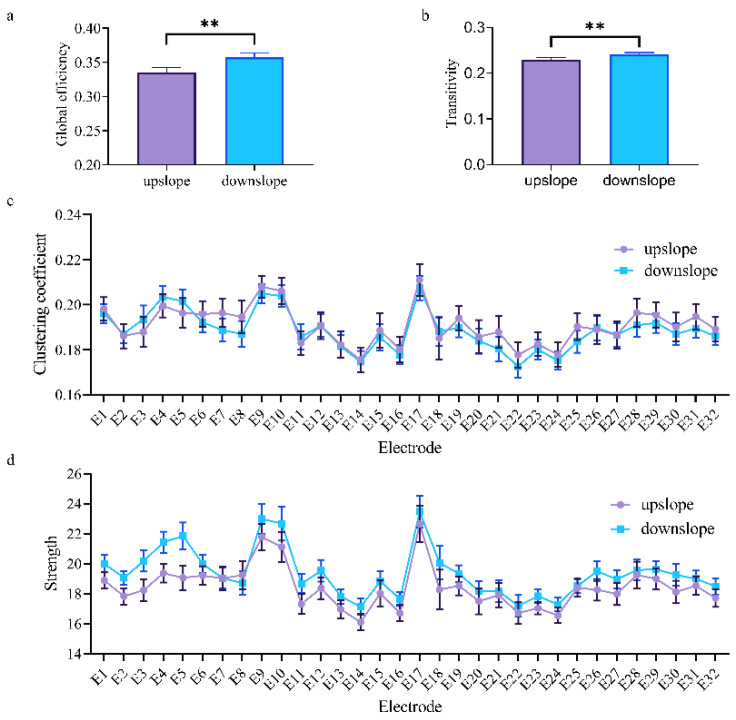
Indexes of the brain network for the two locomotion states in the beta band (13–30 Hz). (**a**,**b**) Global indexes of the brain network, including global efficiency and transitivity. (**c**,**d**) Local indexes of the brain network, including clustering coefficient and strength. All error bars indicate the 95% confidence interval of the mean. ** *p* ≤ 0.01.

**Figure 8:**
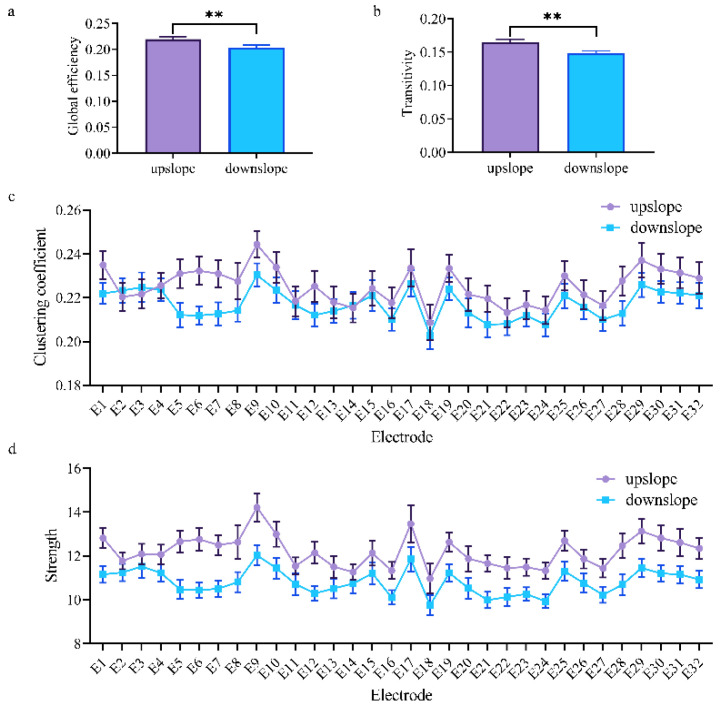
Indexes of the brain network for the two locomotion states in the low gamma band (30–50 Hz). (**a**,**b**) Global indexes of the brain network, including global efficiency and transitivity. (**c**,**d**) Local indexes of the brain network, including clustering coefficient and strength. All error bars indicate the 95% confidence interval of the mean. ** *p* ≤ 0.01.

**Figure 9:**
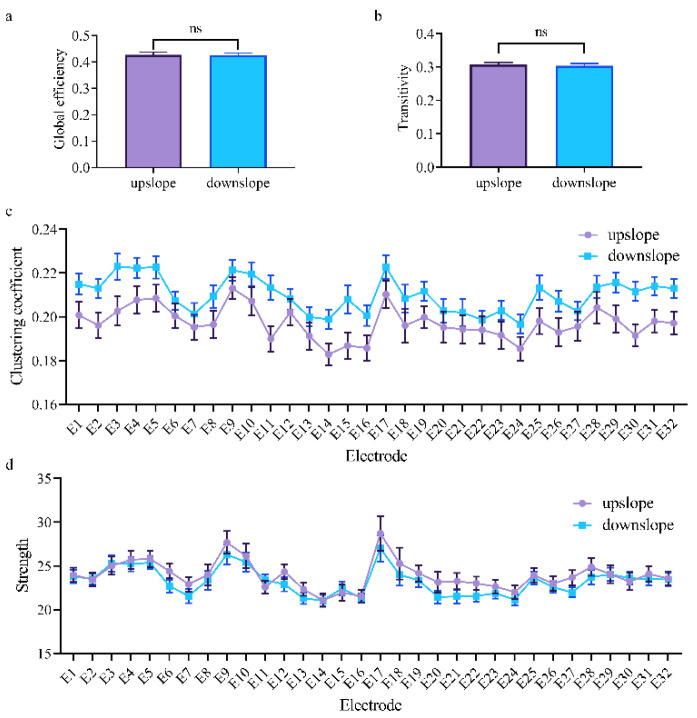
Indexes of the brain network for the two locomotion states in the high gamma band (50–100 Hz). (**a**,**b**) Global indexes of the brain network, including global efficiency and transitivity. (**c**,**d**) Local indexes of the brain network, including clustering coefficient and strength. All error bars indicate the 95% confidence interval of the mean. ns, not significant.

At the network level, the brain network indexes showed striking differences in alpha, beta, and low gamma bands for upslope and downslope walking. By contrast, the global efficiency and transitivity of the brain network in the alpha and low gamma bands were greater in upslope-walking rats than in the downslope-walking ones (Figure 6a,b and Figure 8a,b). In the beta band, the global efficiency and transitivity of the brain network were smaller in the downslope-walking ones than in the upward-walking ones upslope state (Figure 7a,b). Notably, the global efficiency and transitivity of the brain network in the high gamma band showed no significant differences in the upslope-walking rats than in the downslope-walking ones (Figure 9a,b). At the node level, the connectivity indexes also varied with the locomotion states. In the alpha band, the clustering coefficient was significantly greater for the upslope state than for the downward state (Figure 6c), whereas for the node strength, the values increased slightly for the upslope state compared with the downslope state (Figure 6d). In the beta band, no significant differences in the clustering coefficient were observed between the two locomotion states (Figure 7c). Notably, the node strength was significantly greater for the downslope state than the upward one (Figure 7d). The nodes located in the right sensorimotor areas exhibited higher clustering coefficient and strength. In the low gamma band, the clustering coefficients of most nodes were greater when rats walked on the upslope state (Figure 8c). All node strengths were significantly greater for the upslope state than the downslope state (Figure 8d). Notably, in the high gamma band, although no significant differences in global efficiency and transitivity were observed between both states, the clustering coefficient was markedly greater when rats walked on the upslope state than on the downslope state (Figure 9c). No significant difference in node strength was found (Figure 9d). In summary, brain functional directed connectivity in rats markedly varied between both states for each sub-band.

### 3.3. Identifying Locomotion States by Their Brain Functional Directed Connectivity

**Figure 10:**
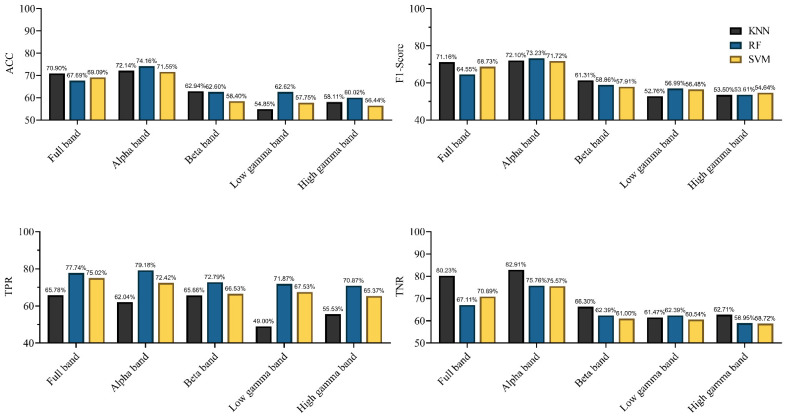
Performances of the different classifiers, characterized by EEG signals in different frequency bands (in terms of Accuracy (ACC), F1-score, true positive ratio (TPR), and true negative rate (TNR)).

In this study, we used three kinds of classification algorithms to detect rat locomotion states and recorded the results for each classifier. To explore the altered connections in different frequency bands when rats were in different locomotion states, the classifiers were applied in the alpha, beta, low gamma, high gamma, and full bands. We recorded the results for the different classifiers in different frequency bands via 10-fold cross-validation. Simultaneously, in evaluating the performance of classification characterized by connectivity, we used EEG signals as features to classify the motion states. The performances of the different classifiers characterized by EEG signals in different frequency bands are shown in Figure 10. The results in Figure 10 shows that different classifiers yielded different results for the same band EEG set. The KNN classifier achieved the highest accuracy of 70.9% (F1-score = 71.16%) in the full frequency band. For the other two classifiers, the classification accuracy of SVM was 69.09% (F1-score = 69.73%), and that of RF was 67.69% (F1-score = 64.55%). For different frequency bands, the same kind of classifier yielded markedly different classification results. The highest classification accuracy was achieved in the alpha band, and the accuracy and F1-score of the three classifiers exceeded 70%. However, this classification result was not satisfactory (F1-scores <80%, according to the definition in the Methods section). The results indicate that using EEG signals as classification features in locomotion state detection cannot achieve satisfactory results. 

**Figure 11:**
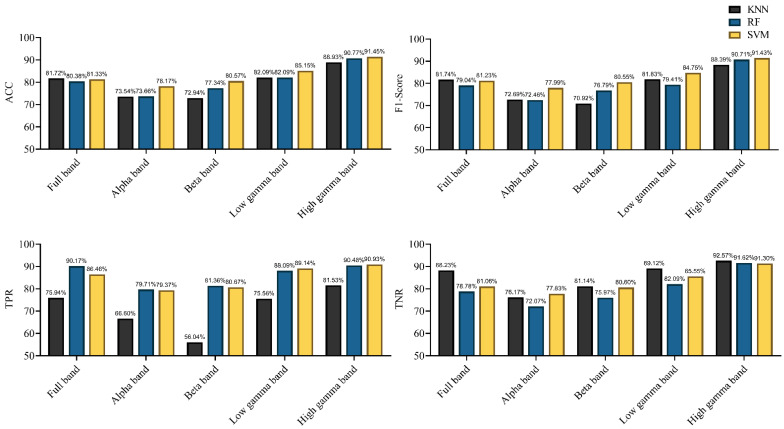
Performance of the different classifiers, characterized by functional directed connectivity in different frequency bands (in terms of ACC, F1-score, TPR, and TNR).

Characterized by the functional directed connectivity in different bands, the performances achieved using different classifiers are shown in Figure 11. All classifiers achieved an accuracy of 80% in the full-frequency band. With the KNN and SVM classifiers, the F1-score surpassed 80%. The performance markedly improved relative to that with the EEG signal as the classifier. Notably, the best classification was achieved using the high gamma band, achieving the highest accuracy (91.45%) (F1-score = 91.43%) with the SVM classifier; even the lowest accuracy, achieved using KNN, also reached 88.93% (F1-score = 88.39%). For other frequency bands, SVM still performed more efficiently. In summary, the results indicate that the connectivity features of the high gamma band contain the best discriminative information with respect to rat locomotion detection. SVM provided the best detection results in most frequency bands, and RF was partly better than KNN.

## 4. Discussion

Although rodents are often used in neuroscience research, our knowledge of brain connectivity properties in walking rats remains limited. In the present study, we investigated the difference in brain functional directed connectivity in rats during different locomotion states and proposed a novel method based on brain functional directed connectivity for locomotion state detection. Results showed that brain functional directed connectivity showed a significant difference in the full-frequency band and each sub-band when the rats were in upslope and downslope locomotion. The classification results also indicated that the connectivity features of the high gamma band contained the best discriminative information with respect to locomotion detection in rats. These findings elucidate the characteristics of neural information interaction between various cortical areas in freely walking rats. 

Consequently, in the full-frequency band, brain functional directed connectivity exhibited an apparent difference between rats walking upslope and downslope (Figure 3). Compared with the rats in downslope locomotion, those in upward locomotion showed stronger connectivity (including large-degree nodes, more connections, and greater weights) (Figure 3a). By contrast, only a small number of connections increased in downslope walking, compared with upslope walking (Figure 3b). The reason could be that, the greater gait length and higher locomotion velocity of rats walking uphill led to more connections between brain regions [14,39]. In addition, for upslope locomotion, the increased edges mostly started from the left motor cortex to other brain areas, whereas for downslope locomotion, the right visual area served as a network hub with numerous outgoing edges to other regions. This occurrence is possible that rats adopt different motion strategies when performing uphill and downhill walking. For upslope walking, in order to move continuously the body mass upward and forward, rats may need to increase muscle strength and improve the speed to ensure that the paws clear uphill obstacles during each swing [40]. Meanwhile, for downslope walking, rats may need to constantly adjust their posture and rely more on proprioception feedback to increase braking force to counteract the external force that accelerates joint movement [41]. Specifically, the brain network characteristics largely varied between upslope and downslope walking (Figure 4), with the former exhibiting higher global efficiency and transitivity than the latter (Figure 4a,b). That is, compared with downslope walking, upward walking required more neural information interactions between various areas of the brain. The clustering coefficient and node strength showed a similar trend (Figure 4c,d). In addition, the nodes located in both the posterior parietal association area and the retrosplenial area showed higher efficiency in information processing. This difference may be attributable to the crucial role of the posterior parietal and retrosplenial cortex in the integration of motor, visual, and spatial information during locomotion [42,43,44]. 

The results further showed that functional directed connectivity in the rat brain presented significant differences for each sub-band between the rats walking upslope and those walking downslope. In the alpha band, the rats in upslope locomotion showed greater brain network connectivity (including large-degree nodes, more connections, and greater weights), compared with the rats in downslope locomotion (Figure 5a). Moreover, the increased stronger connections mainly originated from the left motor cortex to other brain regions. Several studies indicated that alpha band modulation was linked to the degree of volitional engagement, particularly in demanding walking tasks [39,45]. This relation was consistent with the finding that rats need to participate more actively to increase the speed of locomotion and thus remove obstacles to climb uphill. Moreover, the higher global efficiency and transitivity of the brain network in upslope than downslope walking indicated that the rats required more efficient neural information transmission to drive the rapid contraction of the muscles and thus perform uphill locomotion (Figure 6a,b). 

By contrast, in the beta band, the brain network in the rats performing downslope locomotion had more connections (Figure 5f). In addition, the increased edges mainly connected the somatomotor and somatosensory areas. Meanwhile, few connections were increased for the rats in upslope locomotion than those in downslope locomotion (Figure 5b). A previous review suggested that the synchronization or desynchronization of β band activity might indicate the interruption and maintenance of the current movement [46]. Thus, in the current study, the increased connections may reflect the adjustment in the gait of the rats to continue the next phase of walking. Similarly, the brain network exhibited higher global efficiency and transitivity in the downslope-walking than in the upslope-walking rats (Figure 7a,b). This difference suggests that downhill walking required stronger synchronization of beta-band activity to allow more efficient feedback processing (e.g., proprioceptive signals), which is necessary for monitoring the status quo and recalibrating the sensorimotor system [47]. The results for the nodes located in the left sensorimotor areas with greater clustering coefficient and strength verified that the somatosensory cortex is a dominant source of information flow in the beta band [48,49]. 

In the low gamma band, the brain network of the rats in upslope locomotion had more connections, larger-degree nodes, and greater weights (Figure 5c). Prior studies suggested that rhythm in the low gamma band was related to increased cortical computation [50] and reflected the requirements for cortical engagement in the walking task [51]. In the current study, the increased connectivity in the low gamma band may be a sign of increased demand for cortical muscle interactions in the coordination and control of peripheral movements [39]. Compared with upslope walking, downslope walking exhibited significantly lower global efficiency and transitivity (Figure 8a,b). Some studies indicated that low gamma power decreased in walking tasks requiring gait adjustment in response to visual feedback [52]. This finding suggested that rats walking downhill may require more visual information to drive the next movement.

Notably, in the high gamma band, the brain network in rats walking upslope and rats walking downslope both increased abundant connected edges (Figure 5d,h). Compared with other frequency bands, the global efficiency and transitivity of the brain network significantly increased for the two locomotion states (Figure 9a,b). Studies suggested that gamma band oscillations reflected the dynamics of local neuronal groups [53,54] and may reflect more directly the specific details of rats walking. Moreover, a prior study indicated that power fluctuations in the high gamma band were related to the processing of sensory information during movement execution [55]. Thus, in the current study, the increased connectivity of the high gamma band may serve to facilitate kinesthetic feedback from muscles and joints in rats during walking. Although no significant difference in global efficiency and transitivity was found, the clustering coefficient of the nodes was markedly larger in downslope walking than in upslope walking. The reason might be that downhill locomotion required more efficient information processing to accelerate kinesthetic feedback to achieve gait adjustment.

We subsequently determined whether machine learning algorithms can be used to classify locomotion in rats the difference in brain network connectivity. Previous studies suggested that effective connectivity measures carried critical information about the neural properties of cognition and behavior [5]. Moreover, effective connectivity has been used to decode hand joint angles during grasp [12]. Results demonstrated that using functional directed connectivity as classification features for locomotion detection exhibited satisfactory performance (Figure 11). Specifically, the KNN and SVM classifiers achieved an accuracy of 81% (F1-score > 81%) in the full-frequency band. However, the classifiers using EEG signals as classification features failed to achieve the desired effect, even the highest classification accuracy did not exceed 80% (Figure 10). Those results indicated that complex network analysis can be employed in parsing EEG signals to obtain discriminative information features for locomotion detection in rats. Further, the obvious difference in the classification performance was observed in different frequency bands. The highest classification efficiency was observed in the high gamma band, with the highest accuracy reaching 91.45% (F1-score = 91.43%) by using the SVM classifier; even the classifier (KNN) with the lowest accuracy also reached 88.93% (F1-score was 88.39%). The lowest classification efficiency was observed in the alpha and beta bands. Some recent studies suggested that the spectrum power in high gamma band oscillated along with gait cycle phase during human treadmill walking [56]. Moreover, our previous research also suggested that high gamma band activity presented significant difference in different walking task in rats [14]. Those studies indicated that gamma rhythm contained rich motion information, and network analysis can effectively extract the discriminative features among them. That might be why high gamma oscillations could give rise to a better classification rate. Some studies have suggested the use of high gamma signals to decode highly fractionated movements [57], further controlling prosthetic limbs with large degrees of freedom [58,59]. Another study suggested that the intracortical microstimulation based on implanted neuroprosthesis induced the gamma band oscillations in the primary auditory cortex in a similar way as a natural sensory information communication process [60]. All those results showed that the analysis of gamma band signal might be a potential method for improving the accuracy of neuroprosthetic control. The results also showed that different classifiers yielded different results. It was easy to observe that the SVM classifier exhibited the best performance in most frequency bands, and the performance of RF was better than that of KNN. However, the differences between the three classifiers were not that evident in the same frequency band.

## 5. Conclusions

In this study, we revealed the brain functional directed connectivity properties in rats during different walking locomotion states and verified the feasibility of locomotion detection in rats based on brain functional directed connectivity. The results indicated that the functional directed connectivity in the rat brain showed apparent differences in the full frequency band and each sub-band between rats in upslope and downslope walking. Moreover, the use of functional directed connectivity as the classification feature for locomotion detection achieved satisfactory performance. Specifically, the classification results indicated that the connectivity features of the high gamma band contained the best discriminative information with respect to locomotion detection in rats, and the SVM classifier exhibited the best performance. This finding can elucidate rodent walking and help advance the research in neuro-prosthetic limb control in brain–machine interfaces.

## Data Availability

The datasets obtained during the current study are available from the corresponding author on reasonable request.
